# A Hilbert-type fractal integral inequality and its applications

**DOI:** 10.1186/s13660-017-1360-9

**Published:** 2017-04-21

**Authors:** Qiong Liu, Wenbing Sun

**Affiliations:** Department of Science and Information Science, Shaoyang University, Shaoyang, 422000 P.R. China

**Keywords:** fractal set, Hilbert-type fractal integral inequality, weight function

## Abstract

By using thefractal theory and the methods of weight function, a Hilbert-type fractal integral inequality and its equivalent form are given. Their constant factors are proved being the best possible, and their applications are discussed briefly.

## Introduction

If $f, g \geq0$, satisfying $0 < \int_{0}^{\infty} f^{2} ( x ) \,dx <\infty$, $0 < \int_{0}^{\infty} g^{2} ( y ) \,dy <\infty$, then there is the following basic Hilbert-type integral inequality and its equivalent form [1]

1 $$\begin{aligned}& \int_{0}^{\infty} \int_{0}^{\infty} \frac{f ( x ) g(y)}{\max \{ x, y \}} \,dx\,dy< 4 \biggl\{ \int_{0}^{\infty} f^{2} ( x ) \,dx \biggr\} ^{\frac{1}{2}} \biggl\{ \int_{0}^{\infty} g^{2} ( y ) \,dy \biggr\} ^{\frac{1}{2}}, \end{aligned}$$

2 $$\begin{aligned}& \int_{0}^{\infty} \biggl[ \int_{0}^{\infty} \frac{f ( x )}{ \max \{ x, y \}} \,dx \biggr] ^{2} \,dy< 16 \int_{0}^{\infty} f^{2} ( x ) \,dx, \end{aligned}$$

where the constants are optimal. Inequalities (1) and (2) are important in the analysis and partial differential equations [1, 2]. In 2004 and 2006, respectively, (1) and (2) were generalized and improved by introducing an independent parameter *λ* and two parameters $\lambda_{1}$, $\lambda_{2}$ [3, 4].

In recent years, the fractal theory has been developed rapidly, and it has been widely used in the fields of science and engineering. Some researchers have used the fractal theory to discuss and generalize some classical inequalities on fractal sets [5, 6], but the research into the Hilbert-type integral inequality on the fractal set is still not involved. In this paper, by using the fractal theory and the method of weight function to make a meaningful attempt, a Hilbert-type integral inequality and its equivalent form on a fractal set are established.

## Preliminaries

### Definition 2.1

[7]

A non-differentiable function $f: \mathbb{R} \rightarrow \mathbb{R}^{\alpha}$ ($0 <\alpha \leq1$), $x \rightarrow f ({x})$ is called local fractional continuous at $x_{0}$ if for any $\varepsilon > 0$, there exists $\delta >0$ such that $\vert f ( x ) -f( x_{0} ) \vert < \varepsilon^{\alpha}$ whenever $\vert x- x_{0} \vert < \delta$. If $f ({x})$ is local fractional continuous on the interval $( a, b )$, we denote $f ({x})\in C_{\alpha} (a,b)$.

### Definition 2.2

[7]

The local fractional derivative of $f ({x})$ of order *α* ($0 <\alpha\leq1$) at $x_{0}$ is defined by

$$f^{\alpha} ( x_{0} ) = \frac{d^{\alpha} f(x)}{d x^{\alpha}} \bigg|_{x= x_{0}} = \lim_{x\rightarrow x_{0}} \frac{\varGamma (\alpha+1)(f ( x ) -f ( x_{0} ) )}{(x- x_{0} )^{\alpha}}, $$

where $\varGamma ({z} ) = \int_{0}^{\infty} e^{-u} u^{z-1} \, du$ ($z>0$) [8]. If for all $x \in I \subseteq \mathbb{R}$, there exists $f^{ ( k+1 ) \alpha} ( x ) = \mathop{\overbrace{D_{x}^{\alpha} \cdots D_{x}^{\alpha}}}\limits^{k+1} f(x)$, then we denote $f \in D_{ ( k+1 ) \alpha} (I)$, where $k=0,1,2,\ldots$ .

### Lemma 2.1

[9]

*Suppose that* $f ({x})\in C_{\alpha} (a,b)$ *and* $f ({x})\in D_{\alpha} (a,b)$. *Then*, *for* $0 <\alpha \leq1$, *we have an* *α*-*differential form*

$$d^{\alpha} f ( {x} ) = f^{ ( \alpha )} ( x )\, d x^{\alpha}. $$

### Lemma 2.2

[5]

*Let* *I* *be an interval*, $f,g:I \subseteq \mathbb{R}\rightarrow \mathbb{R}^{\alpha}$ ($I^{0}$ *is the interior of* *I*) *such that* $f,g\in D_{\alpha} ( I^{0} )$. *Then the following differentiation rules are valid*:

(i) $\frac{d^{\alpha} (f ( x ) \pm g ( x ) )}{d x^{\alpha}} = f^{ ( \alpha )} (x)\pm g^{ ( \alpha )} (x)$;
(ii) $\frac{d^{\alpha} (f ( x ) g ( x ) )}{ d x^{\alpha}} = f^{ ( \alpha )} ( x ) g ( x ) +f(x) g^{ ( \alpha )} (x)$;
(iii) $\frac{d^{\alpha} \frac{f(x)}{g(x)}}{d x^{\alpha}} = \frac{f^{ ( \alpha )} ( x ) g(x)- f(x)g^{ ( \alpha )} (x)}{g^{2} (x)}$ ($g(x)\neq0$);
(iv) $\frac{d^{\alpha} (Cf ( x ) )}{d x^{\alpha}} =C f^{ ( \alpha )} (x)$, *where* *C* *is a constant*;
(v) *If* *y*(*x*)=(*f*∘*g*)(*x*), *then* $\frac{d^{\alpha} y ( x )}{d x^{\alpha}} = f^{ ( \alpha )} (g ( x ) )( g^{ ( 1 )} (x) )^{\alpha}$.

### Definition 2.3

[7]

Let $f ({x})\in C_{\alpha} (a,b)$. Then the local fractional integral is defined by

$${}_{a} I_{b}^{\alpha} f ( x ) = \frac{1}{\varGamma (\alpha+1)} \int_{a}^{b} f(t) (dt )^{\alpha} = \frac{1}{\varGamma (\alpha+1)} \lim_{\lambda\rightarrow0} \sum_{i=1}^{N} f( t_{i} ) (\Delta t_{i} )^{\alpha}, $$

with $\Delta t_{i} = t_{i} - t_{i-1}$ ($i=1,\ldots,N$) and $\lambda= \max_{1\leq i\leq N} \{\Delta t_{i} \}$, and $a = t_{0} < t_{1} <\cdots< t_{N} =b$ is partition of interval $[ a,b ]$. Here, it follows that ${}_{a} I_{b}^{\alpha} =0$ if $a = b$, ${}_{a} I_{b}^{\alpha} f ( x ) =- {}_{b} I_{a}^{\alpha} f ( x )$ if $a < b$.

### Lemma 2.3

[7]

1. *Suppose that* $f ( x ) = g^{(\alpha)} ( x )\in C_{\alpha} (a,b)$, *then we have*
  $${}_{a} I_{b}^{\alpha} f ( x ) = g ( b ) - g( a ); $$
2. *Suppose that* $f(x),g(x) \in D_{\alpha} (a,b)$, *and* $f^{ ( \alpha )} ( x ), g^{ ( \alpha )} ( x ) \in C_{\alpha} ( a,b )$, *then we have*
  $${}_{a} I_{b}^{\alpha} f ( x ) g^{ ( \alpha )} ( x ) =f ( x ) g ( x ) |_{a}^{b} - {}_{a} I_{b}^{\alpha} f^{ ( \alpha )} ( x ) g ( x ). $$

### Lemma 2.4

[7]

*For* $f ( x ) = x^{\gamma}$ ($\gamma>0$), *we have the following equations*:

$$\begin{aligned}& \frac{d^{\alpha} ( x^{\gamma} )}{d x^{\alpha}} = \frac{\varGamma (1+\gamma)}{ \varGamma (1+\gamma-\alpha)} x^{\gamma-\alpha}; \\& \frac{1}{\varGamma ( \alpha+1 )} \int_{a}^{b} x^{\gamma} (dx )^{\alpha} = \frac{\varGamma ( 1+\gamma )}{\varGamma ( 1+\gamma+\alpha )} \bigl( b^{\gamma+\alpha} - a^{\gamma+\alpha} \bigr). \end{aligned}$$

### Lemma 2.5

[7, 10]

*If* $f,g\ ( \geq0 ) \in C_{\alpha} ( a,b )$, $F,G,h\ (\geq 0) \in C_{\alpha} ( S^{(\beta)} )$, $p>1$, $\frac{1}{p} + \frac{1}{q} =1$, $S^{(\beta)}$ *is a fractal surface*, *then we have*

(i) *Hölder’s inequality on the fractal set*
  $$\begin{aligned}& \frac{1}{\varGamma (\alpha+1)} \int_{a}^{b} f(x)g(x) (dx )^{\alpha} \\& \quad \leq \biggl\{ \frac{1}{\varGamma ( \alpha+1 )} \int_{a}^{b} f^{p} (x) (dx )^{\alpha} \biggr\} ^{\frac{1}{p}} \biggl\{ \frac{1}{ \varGamma ( \alpha+1 )} \int_{a}^{b} g^{q} (x) (dx )^{\alpha} \biggr\} ^{\frac{1}{q}}; \end{aligned}$$
(ii) *Hölder’s weighted inequality on the fractal set*
  $$\begin{aligned}& \frac{1}{\varGamma ^{2} (\alpha+1)} \iint_{S^{(\beta )}} h(x,y)F(x,y)G(x,y) (dx)^{\alpha} (dy)^{\alpha} \\& \quad \leq \biggl\{ \frac{1}{\varGamma ^{2} ( \alpha+1 )} \iint_{S^{(\beta )}} h(x,y)F^{p}(x,y) (dx)^{\alpha} (dy)^{\alpha} \biggr\} ^{\frac{1}{p}} \\& \qquad {}\times\biggl\{ \frac{1}{\varGamma ^{2} ( \alpha+1 )} \iint_{S^{(\beta )}} h(x,y)G^{p}(x,y) (dx)^{\alpha} (dy)^{\alpha} \biggr\} ^{\frac{1}{q}}. \end{aligned}$$

*The inequality keeps the form of equality*, *then there exist constants* *A* *and* *B* *such that they are not all zero and* $AF^{p} ( x,y ) =B G^{q} (x,y)$ *a*.*e*. *on* $S^{(\beta)}$.

### Lemma 2.6

*Suppose that* $p > 1$, $\frac{1}{p} + \frac{1}{q} =1$, $0 <\alpha \leq1$, *and weight functions are defined by*

$$\begin{aligned}& \omega ( \alpha, p,x ) := \frac{1}{\varGamma ( \alpha+1 )} \int_{0}^{\infty} \frac{1}{\max \{ x^{\alpha}, y^{\alpha} \} } \frac{y^{- \frac{\alpha}{2}}}{ x^{- \frac{p\alpha}{2q}}} ( dy )^{\alpha},\quad x\in ( 0, +\infty ), \\& \omega ( \alpha, q,y ) := \frac{1}{\varGamma ( \alpha+1 )} \int_{0}^{\infty} \frac{1}{\max \{ x^{\alpha}, y^{\alpha} \} } \frac{x^{- \frac{\alpha}{2}}}{ y^{- \frac{q\alpha}{2p}}} ( dx )^{\alpha},\quad y\in ( 0, +\infty ). \end{aligned}$$

*Then*

$$\omega ( \alpha, p,x ) =\eta ( \alpha ) x^{\frac{\alpha}{2} (p-2)},\qquad \omega ( \alpha, q,y ) =\eta ( \alpha ) y^{\frac{\alpha}{2} (q-2)}, $$

*where*

3 $$ \eta ( \alpha ) = \frac{2^{\alpha+1}}{\varGamma (1+\alpha)}. $$

### Proof

Set $\frac{y}{x} =u$, then $( dy)^{\alpha} = x^{\alpha} ( du )^{\alpha}$. Note the following exchange integral, let $u= \frac{1}{t}$, and $\sqrt{u} =s$, we have $(du)^{\alpha} = - t^{-2\alpha} ( dt )^{\alpha}$ and $u^{- \frac{\alpha}{2}} (du)^{\alpha} = 2^{\alpha} (ds)^{\alpha}$. Then we have

$$\begin{aligned} \omega ( \alpha, p,x ) =& \frac{1}{\varGamma ( \alpha+1 )} \int_{0}^{\infty} \frac{1}{\max \{ x^{\alpha}, y^{\alpha} \} } \frac{y^{- \frac{\alpha}{2}}}{x^{- \frac{p\alpha}{2q}}} ( dy )^{\alpha} \\ =&x^{\frac{\alpha}{2} ( p-2 )} \frac{1}{\varGamma ( \alpha+1 )} \int_{0}^{\infty} \frac{\max\{1,u^{\alpha}\}}{\max \{ x^{\alpha}, y^{\alpha} \} } u^{- \frac{\alpha}{2}} ( du )^{\alpha} \\ =&x^{\frac{\alpha}{2} ( p-2 )} \frac{1}{\varGamma ( \alpha+1 )} \biggl[ \int_{0}^{1} u^{- \frac{\alpha}{2}} ( du )^{\alpha} + \int_{1}^{\infty} u^{- \frac{3\alpha}{2}} ( du )^{\alpha} \biggr] \\ =&x^{\frac{\alpha}{2} ( p-2 )} \frac{1}{\varGamma ( \alpha+1 )} \biggl[ \int_{0}^{1} u^{- \frac{\alpha}{2}} ( du )^{\alpha} + \int_{0}^{1} t^{- \frac{\alpha}{2}} ( dt )^{\alpha} \biggr] \\ =&x^{\frac{\alpha}{2} ( p-2 )} \biggl( \frac{2}{\varGamma ( \alpha+1 )} \int_{0}^{1} u^{- \frac{\alpha}{2}} ( du )^{\alpha} \biggr) \\ =&x^{\frac{\alpha}{2} ( p-2 )} \biggl( \frac{2^{\alpha+1}}{ \varGamma ( \alpha+1 )} \int_{0}^{1} ( ds )^{\alpha} \biggr) \\ =& \frac{2^{\alpha+1}}{\varGamma ( 1+\alpha )} x^{\frac{\alpha}{2} ( p-2 )} \\ =&\eta ( \alpha ) x^{\frac{\alpha}{2} (p-2)}. \end{aligned}$$

Similarly, we obtain $\omega ( \alpha, q,y ) =\eta ( \alpha ) y^{\frac{\alpha}{2} (q-2)}$. □

### Lemma 2.7

*Suppose that* $p > 1$, $\frac{1}{p} + \frac{1}{q} =1$, $0 <\alpha \leq1$, *and* $\varepsilon > 0$ *is small enough*, *let us define the real functions as follows*:

$$\overline{f} ( x ) = \left \{ \textstyle\begin{array}{l@{\quad}l} 0, &x\in(0,1), \\ x^{- \frac{\alpha}{2} - \frac{\alpha\varepsilon}{p}},& x\in[1,\infty), \end{array}\displaystyle \right . \qquad \overline{g} ( y ) = \left \{ \textstyle\begin{array}{l@{\quad}l} 0,& y\in(0,1), \\ x^{- \frac{\alpha}{2} - \frac{\alpha\varepsilon}{q}},& y\in[1,\infty), \end{array}\displaystyle \right . $$

*then we have*

4 $$\begin{aligned}& \overline{J} \cdot\varepsilon^{\alpha} = \bigl[ {}_{0} I_{\infty}^{\alpha} \bigl( x^{\frac{\alpha}{2} ( p-2 )} \overline{f}^{p} ( x ) \bigr) \bigr]^{\frac{1}{p}} \bigl[ {}_{0} I_{\infty}^{\alpha} \bigl( y^{\frac{\alpha}{2} ( q-2 )} \overline{g}^{q} ( y ) \bigr) \bigr]^{\frac{1}{q}} \cdot\varepsilon^{\alpha} = \frac{1}{\varGamma ( \alpha+1 )}, \end{aligned}$$

5 $$\begin{aligned}& \overline{h} \cdot\varepsilon^{\alpha} = {}_{0} I_{\infty}^{\alpha} \biggl[ {}_{0} I_{\infty}^{\alpha} \frac{\overline{f} (x) \overline{g} (y)}{\max \{ x^{\alpha}, y^{\alpha} \} } \biggr] \cdot\varepsilon^{\alpha} > \frac{\eta ( \alpha )}{\varGamma ( \alpha+1 )} \bigl( 1-{o} ( 1 ) \bigr)\quad \bigl( \varepsilon\rightarrow 0^{+} \bigr). \end{aligned}$$

### Proof

Note the properties of local fractal space [5, 7]: $( a+b )^{\alpha} = a^{\alpha} + b^{\alpha}$ and $( -a )^{\alpha} =- a^{\alpha}$, we easily obtain

$$\begin{aligned} \overline{J} \cdot\varepsilon^{\alpha} =& \bigl[ {}_{0} I_{\infty}^{\alpha} \bigl( x^{\frac{\alpha}{2} ( p-2 )} \overline{f}^{p} ( x ) \bigr) \bigr]^{\frac{1}{p}} \bigl[ {}_{0} I_{\infty}^{\alpha} \bigl( y^{\frac{\alpha}{2} ( q-2 )} \overline{g}^{q} ( y ) \bigr) \bigr]^{\frac{1}{q}} \cdot\varepsilon^{\alpha} \\ =& \bigl[ {}_{1} I_{\infty}^{\alpha} \bigl( x^{-\alpha(1+\varepsilon)} \bigr) \bigr]^{\frac{1}{p}} \bigl[ {}_{1} I_{\infty}^{\alpha} \bigl( y^{-\alpha(1+\varepsilon)} \bigr) \bigr]^{\frac{1}{q}} \cdot\varepsilon^{\alpha} = \frac{1}{\varGamma ( \alpha+1 )}. \end{aligned}$$

Let $t^{\frac{1}{2} - \frac{\varepsilon}{q}} =u$, $x^{- \frac{1}{2} + \frac{\varepsilon}{q}} =v$, and from $( \frac{1}{2} - \frac{\varepsilon}{q} )^{\alpha} t^{- \frac{\alpha}{2} - \frac{\alpha\varepsilon}{q}} ( dt )^{\alpha} = ( du )^{\alpha}$, $- ( \frac{1}{2} - \frac{\varepsilon}{q} )^{\alpha} x^{- \frac{3\alpha}{2} + \frac{\alpha\varepsilon}{q}} ( dx )^{\alpha} = ( dv )^{\alpha}$, we write

$$\begin{aligned} {}_{1} I_{\infty}^{\alpha} \bigl( x^{-\alpha} \bigr) \cdot {}_{0} I_{\frac{1}{x}}^{\alpha} \bigl( t^{- \frac{\alpha}{2} - \frac{\alpha\varepsilon}{q}} \bigr) =& \frac{1}{\varGamma ^{2} ( \alpha+1 )} \biggl( \int_{1}^{\infty} x^{-\alpha} ( dx )^{\alpha} \biggr) \biggl( \int_{0}^{\frac{1}{x}} t^{- \frac{\alpha}{2} - \frac{\alpha\varepsilon}{q}} ( dt )^{\alpha} \biggr) \\ =& \frac{1}{ ( \frac{1}{2} - \frac{\varepsilon}{q} )^{\alpha} \varGamma ^{2} ( \alpha+1 )} \biggl( \int_{1}^{\infty} x^{-\alpha} ( dx )^{\alpha} \biggr) \biggl( \int_{0}^{x^{- \frac{1}{2} + \frac{\varepsilon}{q}}} ( dv )^{\alpha} \biggr) \\ =& \frac{1}{ ( \frac{1}{2} - \frac{\varepsilon}{q} )^{\alpha} \varGamma ^{2} ( \alpha+1 )} \int_{1}^{\infty} x^{- \frac{3\alpha}{2} + \frac{\alpha\varepsilon}{q}} ( dx )^{\alpha} \\ =& - \frac{1}{ ( \frac{1}{2} - \frac{\varepsilon}{q} )^{2\alpha} \varGamma ^{2} ( \alpha+1 )} \int_{1}^{0} ( dv )^{\alpha} \\ =& \frac{1}{ ( \frac{1}{2} - \frac{\varepsilon}{q} )^{2\alpha} \varGamma ^{2} ( \alpha+1 )}. \end{aligned}$$

Further, let $\frac{y}{x} =t$, and by Lemma 2.6, we have

$$\begin{aligned} \overline{h} \cdot\varepsilon^{\alpha} =& {}_{0} I_{\infty}^{\alpha} \biggl[ {}_{0} I_{\infty}^{\alpha} \frac{\overline{f} ( x ) \overline{g} ( y )}{\max \{ x^{\alpha}, y^{\alpha} \} } \biggr] \cdot\varepsilon^{\alpha} \\ =& {}_{1} I_{\infty}^{\alpha} \biggl[ \bigl( x^{- \frac{\alpha}{2} - \frac{\alpha\varepsilon}{p}} \bigr) {}_{1} I_{\infty}^{\alpha} \biggl( \frac{y^{- \frac{\alpha}{2} - \frac{\alpha\varepsilon}{q}}}{\max \{ x^{\alpha}, y^{\alpha} \} } \biggr) \biggr] \cdot\varepsilon^{\alpha} \\ =& \bigl[ {}_{1} I_{\infty}^{\alpha} \bigl( x^{-\alpha(1+\varepsilon)} \bigr) \bigr] \biggl[ {}_{\frac{1}{x}} I_{\infty}^{\alpha} \biggl( \frac{t^{- \frac{\alpha}{2} - \frac{\alpha\varepsilon}{q}}}{\max \{ 1, t^{\alpha} \} } \biggr) \biggr] \cdot \varepsilon^{\alpha} \\ =& \bigl[ {}_{1} I_{\infty}^{\alpha} \bigl( x^{-\alpha(1+\varepsilon)} \bigr) \bigr] \\ &{}\times\biggl[ {}_{0} I_{1}^{\alpha} \biggl( \frac{t^{- \frac{\alpha}{2} - \frac{\alpha\varepsilon}{q}}}{\max \{ 1, t^{\alpha} \} } \biggr) + {}_{1} I_{\infty}^{\alpha} \biggl( \frac{t^{- \frac{\alpha}{2} - \frac{\alpha\varepsilon}{q}}}{\max \{ 1, t^{\alpha} \} } \biggr) - {}_{0} I_{\frac{1}{x}}^{\alpha} \biggl( \frac{t^{- \frac{\alpha}{2} - \frac{\alpha\varepsilon}{q}}}{\max \{ 1, t^{\alpha} \} } \biggr) \biggr] \cdot \varepsilon^{\alpha} \\ =& \bigl[ {}_{1} I_{\infty}^{\alpha} \bigl( x^{-\alpha(1+\varepsilon)} \bigr) \bigr] \biggl[ {}_{0} I_{1}^{\alpha} \bigl( t^{- \frac{\alpha}{2} - \frac{\alpha\varepsilon}{q}} \bigr)+ {}_{1} I_{\infty}^{\alpha} \bigl( t^{- \frac{3\alpha}{2} - \frac{\alpha\varepsilon}{q}} \bigr)- {}_{0} I_{\frac{1}{x}}^{\alpha} \biggl( \frac{t^{- \frac{\alpha}{2} - \frac{\alpha\varepsilon}{q}}}{\max \{ 1, t^{\alpha} \} } \biggr) \biggr] \cdot \varepsilon^{\alpha} \\ =& \bigl[ {}_{1} I_{\infty}^{\alpha} \bigl( x^{-\alpha(1+\varepsilon)} \bigr) \bigr] \bigl[ {}_{0} I_{1}^{\alpha} \bigl( t^{- \frac{\alpha}{2} - \frac{\alpha\varepsilon}{q}} \bigr)+ {}_{0} I_{1}^{\alpha} \bigl( t^{- \frac{\alpha}{2} + \frac{\alpha\varepsilon}{q}} \bigr)- {}_{0} I_{\frac{1}{x}}^{\alpha} \bigl( t^{- \frac{\alpha}{2} - \frac{\alpha\varepsilon}{q}} \bigr) \bigr] \cdot \varepsilon^{\alpha} \\ =& \frac{1}{ ( \frac{1}{2} - \frac{\varepsilon}{q} )^{\alpha} \varGamma ^{2} ( \alpha+1 )} + \frac{1}{ ( \frac{1}{2} + \frac{\varepsilon}{q} )^{\alpha} \varGamma ^{2} ( \alpha+1 )} - {}_{1} I_{\infty}^{\alpha} \bigl( x^{-\alpha(1+\varepsilon)} \bigr) \cdot {}_{0} I_{\frac{1}{x}}^{\alpha} \bigl( t^{- \frac{\alpha}{2} - \frac{\alpha\varepsilon}{q}} \bigr) \cdot \varepsilon^{\alpha} \\ =& \frac{\eta ( \alpha )}{\varGamma ( \alpha+1 )} + o_{1} ( 1 ) - {}_{1} I_{\infty}^{\alpha} \bigl( x^{-\alpha(1+\varepsilon)} \bigr) \cdot {}_{0} I_{\frac{1}{x}}^{\alpha} \bigl( t^{- \frac{\alpha}{2} - \frac{\alpha\varepsilon}{q}} \bigr) \cdot \varepsilon^{\alpha} \\ >& \frac{\eta ( \alpha )}{\varGamma ( \alpha+1 )} + o_{1} ( 1 ) - {}_{1} I_{\infty}^{\alpha} \bigl( x^{-\alpha} \bigr) \cdot {}_{0} I_{\frac{1}{x}}^{\alpha} \bigl( t^{- \frac{\alpha}{2} - \frac{\alpha\varepsilon}{q}} \bigr) \cdot \varepsilon^{\alpha} \\ =& \frac{\eta ( \alpha )}{\varGamma ( \alpha+1 )} + o_{1} ( 1 ) - \frac{\varepsilon^{\alpha}}{ ( \frac{1}{2} - \frac{\varepsilon}{q} )^{2\alpha} \varGamma ^{2} ( \alpha+1 )} \\ =& \frac{\eta ( \alpha )}{\varGamma ( \alpha+1 )} \bigl( 1-{o} ( 1 ) \bigr) \quad \bigl( \varepsilon \rightarrow 0^{+} \bigr). \end{aligned}$$

 □

## Main results and applications

Introducing the mark: ${}_{0} I_{\infty}^{\alpha} [ {}_{0} I_{\infty}^{\alpha} F(x,y) ] = \frac{1}{\varGamma ^{2} ( \alpha+1 )} \int_{0}^{\infty} \int_{0}^{\infty} F(x,y) ( dx )^{\alpha} ( dy )^{\alpha}$ (see [7]).

### Theorem 3.1

*If* $p > 1$, $\frac{1}{p} + \frac{1}{q} =1$, $0 <\alpha \leq1$, $f,g\ (>0)\in C_{\alpha} (0, \infty)$, *and* $0 < {}_{0} I_{\infty}^{\alpha} ( x^{\frac{\alpha}{2} ( p-2 )} f^{p} (x) ) <\infty$, $0 < {}_{0} I_{\infty}^{\alpha} ( y^{\frac{\alpha}{2} ( q-2 )} g^{q} (y) ) <\infty$, *then*

6 $$ {}_{0} I_{\infty}^{\alpha} \biggl[ {}_{0} I_{\infty}^{\alpha} \frac{f(x)g(y)}{\max \{ x^{\alpha}, y^{\alpha} \} } \biggr] < \eta ( \alpha ) \bigl\{ {}_{0} I_{\infty}^{\alpha} \bigl( x^{\frac{\alpha}{2} ( p-2 )} f^{p} ( x ) \bigr) \bigr\} ^{\frac{1}{p}} \bigl\{ {}_{0} I_{\infty}^{\alpha} \bigl( y^{\frac{\alpha}{2} ( q-2 )} g^{q} ( y ) \bigr) \bigr\} ^{\frac{1}{q}}, $$

*where the constant factor* $\eta ( \alpha )$ *defined in* (3) *is the best possible*.

### Proof

By Hölder’s weighted inequality on the fractal set and Lemma 2.6, we obtain

7 $$\begin{aligned} {}_{0} I_{\infty}^{\alpha} \biggl[ {}_{0} I_{\infty}^{\alpha} \frac{f(x)g(y)}{\max \{ x^{\alpha}, y^{\alpha} \} } \biggr] =& \frac{1}{\varGamma ^{2} ( \alpha+1 )} \int_{0}^{\infty} \int_{0}^{\infty} \frac{f(x)g(y)}{\max \{ x^{\alpha}, y^{\alpha} \} } ( dx )^{\alpha} ( dy )^{\alpha} \\ =& \frac{1}{\varGamma ^{2} ( \alpha+1 )} \int_{0}^{\infty} \int_{0}^{\infty} \frac{f(x)g(y)}{\max \{ x^{\alpha}, y^{\alpha} \} } \biggl[ \frac{y^{- \frac{\alpha}{2p}}}{x^{- \frac{\alpha}{2q}}} \biggr] \biggl[ \frac{x^{- \frac{\alpha}{2q}}}{y^{- \frac{\alpha}{2p}}} \biggr] ( dx )^{\alpha} ( dy )^{\alpha} \\ \leq& \biggl\{ \frac{1}{\varGamma ^{2} ( \alpha+1 )} \int_{0}^{\infty} \int_{0}^{\infty} \frac{f(x)g(y)}{\max \{ x^{\alpha}, y^{\alpha} \} } \frac{y^{- \frac{\alpha}{2}}}{ x^{- \frac{p\alpha}{2q}}} ( dx )^{\alpha} ( dy )^{\alpha} \biggr\} ^{\frac{1}{p}} \\ &{}\times\biggl\{ \frac{1}{\varGamma ^{2} ( \alpha+1 )} \int_{0}^{\infty} \int_{0}^{\infty} \frac{f(x)g(y)}{\max \{ x^{\alpha}, y^{\alpha} \} } \frac{x^{- \frac{\alpha}{2}}}{y^{- \frac{q\alpha}{2p}}} ( dx )^{\alpha} ( dy )^{\alpha} \biggr\} ^{\frac{1}{q}} \\ =& \biggl\{ \frac{1}{\varGamma ^{2} ( \alpha+1 )} \int_{0}^{\infty} \omega(\alpha,p,x) f^{p} (x) ( dx )^{\alpha} \biggr\} ^{\frac{1}{p}} \\ &{}\times\biggl\{ \frac{1}{\varGamma ^{2} ( \alpha+1 )} \int_{0}^{\infty} \int_{0}^{\infty} \omega(\alpha,q,y) g^{q} (y) ( dy )^{\alpha} \biggr\} ^{\frac{1}{q}} \\ =&\eta ( \alpha ) \biggl\{ \frac{1}{\varGamma ( \alpha+1 )} \int_{0}^{\infty} x^{\frac{\alpha}{2} (p-2)} f^{p} (x) ( dx )^{\alpha} \biggr\} ^{\frac{1}{p}} \\ &{}\times\biggl\{ \frac{1}{\varGamma ( \alpha+1 )} \int_{0}^{\infty} y^{\frac{\alpha}{2} (q-2)} g^{q} (y) ( dy )^{\alpha} \biggr\} ^{\frac{1}{q}} \\ =&\eta ( \alpha ) \bigl\{ {}_{0} I_{\infty}^{\alpha} \bigl( x^{\frac{\alpha}{2} ( p-2 )} f^{p} ( x ) \bigr) \bigr\} ^{\frac{1}{p}} \bigl\{ {}_{0} I_{\infty}^{\alpha} \bigl( y^{\frac{\alpha}{2} ( q-2 )} g^{q} ( y ) \bigr) \bigr\} ^{\frac{1}{q}}. \end{aligned}$$

Now assume that equality holds in (7), there exist two nonzero constants *A* and *B* such that $A \frac{y^{- \frac{\alpha}{2}}}{x^{- \frac{p\alpha}{2q}}} f^{p} ( x )= B \frac{x^{- \frac{\alpha}{2}}}{y^{- \frac{q\alpha}{2p}}}$ a.e. in $(0,\infty)\times(0,\infty)$, then there is constant $C \neq0$ such that $Ax^{\frac{\alpha}{2} ( p-2 )} f^{p} ( x ) = By^{\frac{\alpha}{2} ( q-2 )} g^{q} ( y ) =C$ a.e. in $(0,\infty)\times(0,\infty)$. Assuming that $A \neq0$, we have $x^{\frac{\alpha}{2} ( p-2 )} f^{p} ( x ) = \frac{C}{ A}$ a.e. in $(0,\infty)$. Because $\frac{1}{\varGamma ( \alpha+1 )} \int_{0}^{\infty} \frac{C}{A} ( dx )^{\alpha} = \frac{C}{A\varGamma ( \alpha+1 )} x^{\alpha} |_{0}^{\infty}$ is diffuse, which contradicts the fact that $0 < {}_{0} I_{\infty}^{\alpha} ( x^{\frac{\alpha}{2} ( p-2 )} f^{p} (x) ) <\infty$, thus inequality (7) is strict.

If the constant factor $\eta ( \alpha )$ in (6) is not optimal, then there exists positive $K<\eta ( \alpha )$ such that inequality (6) is still valid if we replace $\eta ( \alpha )$ by *K*. Hence by (4) and (5), we have $\eta ( \alpha ) ( 1-{o} ( 1 ) ) < K$.

Letting $\varepsilon\rightarrow 0^{+}$, we get $K\geq\eta ( \alpha )$, which contradicts the fact that $K<\eta ( \alpha )$, therefore $\eta ( \alpha )$ in (6) is the best possible. □

### Theorem 3.2

*Under the conditions of Theorem* 3.1, *we have*

8 $$ {}_{0} I_{\infty}^{\alpha} \biggl\{ y^{\frac{\alpha(2-q)}{2(q-1)}} \biggl[ {}_{0} I_{\infty}^{\alpha} \frac{f(x)}{\max \{ x^{\alpha}, y^{\alpha} \} } \biggr]^{p} \biggr\} < \eta^{p} ( \alpha ) {}_{0} I_{\infty}^{\alpha} \bigl( x^{\frac{\alpha}{2} ( p-2 )} f^{p} (x) \bigr), $$

*where the constant factor* $\eta^{p} ( \alpha )$ *is the best possible*, *and inequality* (8) *is equivalent to inequality* (6).

### Proof

Define $[ f(x) ]_{n} {:=} \min \{ n, f(x) \}$. Since $0 < {}_{0} I_{\infty}^{\alpha} ( x^{\frac{\alpha}{2} ( p-2 )} f^{p} (x) ) <\infty$, there exists $n_{0} \in \mathbb{N}$ such that $0 < {}_{\frac{1}{n}} I_{n}^{\alpha} ( x^{\frac{\alpha}{2} ( p-2 )} [ f(x) ]_{n}^{p} ) <\infty$ ($n\geq n_{0} $). Setting $g_{n} (y) := y^{\frac{\alpha ( 2-q )}{2 ( q-1 )}} [ {}_{\frac{1}{n}} I_{n}^{\alpha} \frac{ [ f ( x ) ]_{n}}{\max \{ x^{\alpha}, y^{\alpha} \}} ]^{\frac{p}{q}}$ ($\frac{1}{n} < y< n$, $n\geq n_{0}$), when $n\geq n_{0}$, by (6), we find

9 $$\begin{aligned} \begin{aligned}[b] 0 &< {}_{\frac{1}{n}} I_{n}^{\alpha} \bigl( y^{\frac{\alpha}{2} ( q-2 )} g_{n}^{q} (y) \bigr) \\ &= \frac{1}{\varGamma ( \alpha+1 )} \int_{\frac{1}{n}}^{n} y^{\frac{\alpha}{2} ( q-2 )} g_{n}^{q-1} (y ) g_{n} (y) ( dy )^{\alpha} \\ &= \frac{1}{\varGamma ( \alpha+1 )} \int_{\frac{1}{n}}^{n} {}_{\frac{1}{n}} I_{n}^{\alpha} \biggl( \frac{ [ f ( x ) ]_{n}}{\max \{ x^{\alpha}, y^{\alpha} \} } \biggr)y^{\frac{\alpha ( 2-q )}{2 ( q-1 )}} \biggl[ {}_{\frac{1}{n}} I_{n}^{\alpha} \frac{ [ f ( x ) ]_{n}}{\max \{ x^{\alpha}, y^{\alpha} \} } \biggr]^{\frac{p}{q}} ( dy )^{\alpha} \\ &= \frac{1}{\varGamma ^{2} ( \alpha+1 )} \int_{\frac{1}{n}}^{n} \int_{\frac{1}{n}}^{n} \frac{ [ f(x) ]_{n} g_{n} (y)}{\max \{ x^{\alpha}, y^{\alpha} \} } ( dx )^{\alpha} ( dy )^{\alpha} \\ &< \eta ( \alpha ) \bigl\{ {}_{\frac{1}{n}} I_{n}^{\alpha} \bigl( x^{\frac{\alpha}{2} ( p-2 )} \bigl[ f ( x ) \bigr]_{n}^{p} \bigr) \bigr\} ^{\frac{1}{p}} \bigl\{ {}_{\frac{1}{n}} I_{n}^{\alpha} \bigl( y^{\frac{\alpha}{2} ( q-2 )} g_{n}^{q} ( y ) \bigr) \bigr\} ^{\frac{1}{q}}. \end{aligned} \end{aligned}$$

Moreover, by (9) we have

10 $$\begin{aligned} 0 < & {}_{\frac{1}{n}} I_{n}^{\alpha} \bigl( y^{\frac{\alpha}{2} ( q-2 )} g_{n}^{q} (y) \bigr) = {}_{\frac{1}{n}} I_{n}^{\alpha} \biggl\{ y^{\frac{\alpha ( 2-q )}{2 ( q-1 )}} \biggl[ {}_{\frac{1}{n}} I_{n}^{\alpha} \frac{ [ f ( x ) ]_{n}}{\max \{ x^{\alpha}, y^{\alpha} \} } \biggr]^{p} \biggr\} \\ < & \eta^{p} ( \alpha ) {}_{\frac{1}{n}} I_{n}^{\alpha} \bigl( x^{\frac{\alpha}{2} ( p-2 )} \bigl[ f(x) \bigr]_{n}^{p} \bigr) < \infty. \end{aligned}$$

For $n\rightarrow\infty$, it follows that $0 < {}_{0} I_{\infty}^{\alpha} ( y^{\frac{\alpha}{2} ( q-2 )} g_{\infty}^{q} (y) ) <\infty$, and $0 < {}_{0} I_{\infty}^{\alpha} ( x^{\frac{\alpha}{2} ( p-2 )} f^{p} (x) ) <\infty$, by (6), both (9) and (10) still keep the form of strict inequalities. Hence we have inequality (8).

On the other hand, by Hölder’s inequality on the fractal set and (8), we find

$$\begin{aligned}& {}_{0} I_{\infty}^{\alpha} \biggl[ {}_{0} I_{\infty}^{\alpha} \frac{f(x)g(y)}{\max \{ x^{\alpha}, y^{\alpha} \} } \biggr] \\& \quad = \frac{1}{\varGamma ^{2} ( \alpha+1 )} \int_{0}^{\infty} \int_{0}^{\infty} \frac{f(x)g(y)}{\max \{ x^{\alpha}, y^{\alpha} \} } ( dx )^{\alpha} ( dy )^{\alpha} \\& \quad = \frac{1}{\varGamma ( \alpha+1 )} \int_{0}^{\infty} \biggl[ y^{\frac{\alpha ( 2-q )}{2p ( q-1 )}} \frac{1}{ \varGamma ( \alpha+1 )} \int_{0}^{\infty} \frac{f(x)}{\max \{ x^{\alpha}, y^{\alpha} \} } ( dx )^{\alpha} \biggr] \bigl[ y^{\frac{\alpha ( 2-q )}{2p ( q-1 )}} g(y) \bigr] ( dy )^{\alpha} \\& \quad \leq \biggl\{ \frac{1}{\varGamma ( \alpha+1 )} \int_{0}^{\infty} y^{\frac{\alpha ( 2-q )}{2 ( q-1 )}} \biggl[ \frac{1}{ \varGamma ( \alpha+1 )} \int_{0}^{\infty} \frac{f(x)}{\max \{ x^{\alpha}, y^{\alpha} \} } ( dx )^{\alpha} \biggr]^{p} ( dy )^{\alpha} \biggr\} ^{\frac{1}{p}} \\& \qquad {}\times\biggl\{ \frac{1}{\varGamma ( \alpha+1 )} \int_{0}^{\infty} y^{\frac{\alpha}{2} ( q-2 )} g^{q} ( y ) ( dy )^{\alpha} \biggr\} ^{\frac{1}{q}} \\& \quad < \eta ( \alpha ) \bigl\{ {}_{0} I_{\infty}^{\alpha} \bigl( x^{\frac{\alpha}{2} ( p-2 )} f^{p} ( x ) \bigr) \bigr\} ^{\frac{1}{p}} \bigl\{ {}_{0} I_{\infty}^{\alpha} \bigl( y^{\frac{\alpha}{2} ( q-2 )} g^{q} ( y ) \bigr) \bigr\} ^{\frac{1}{q}}. \end{aligned}$$

The above inequality is (6), therefore inequality (8) is equivalent to inequality (6).

If the constant factor in (8) is not optimal, then by (8) we can get a contradiction that the constant factor in (6) is not the optimal too. Thus the constant factor $\eta ^{p} ( \alpha )$ in (8) is the best possible. □

## Simple applications

Selecting *α* values in (6) and (8), and using mathematics software to calculate, some Hilbert-type fractional integral inequalities and their equivalent forms are obtained.

### Example 1

Letting $\alpha =1$, $p = q =2$, to calculate formula (3), we get $\eta ( 1 ) =4$, then we obtain inequalities (1) and (2).

### Example 2

Letting $\alpha =0.5$, $p = q =2$, to calculate formula (3), we get $\eta ( 0.5 ) =4 \sqrt{\frac{2}{\pi}}$. Suppose that $f,g \ ( >0 ) \in C_{0.5} ( 0, \infty )$, $0 < {}_{0} I_{\infty}^{0.5} ( f^{2} ( x ) )<\infty$, $0 < {}_{0} I_{\infty}^{0.5} ( g^{2} ( y ) ) <\infty$, then we have the following equivalence inequalities:

11 $$\begin{aligned}& {}_{0} I_{\infty}^{0.5} \biggl[ {}_{0} I_{\infty}^{0.5} \frac{f(x)g(y)}{\max \{ \sqrt{x}, \sqrt{y} \} } \biggr] < 4 \sqrt{ \frac{2}{\pi}} \bigl\{ {}_{0} I_{\infty}^{0.5} \bigl(f^{2} ( x ) \bigr) \bigr\} ^{\frac{1}{2}} \bigl\{ {}_{0} I_{\infty}^{0.5} \bigl( g^{2} ( y ) \bigr) \bigr\} ^{\frac{1}{2}}, \end{aligned}$$

12 $$\begin{aligned}& {}_{0} I_{\infty}^{0.5} \biggl[ {}_{0} I_{\infty}^{0.5} \frac{f(x)}{\max \{ \sqrt{x}, \sqrt{y} \} } \biggr]^{2} < \frac{32}{ \pi} {}_{0} I_{\infty}^{0.5} \bigl( f^{2} ( x ) \bigr), \end{aligned}$$

where the constant factors $4 \sqrt{\frac{2}{\pi}}$, $\frac{32}{\pi}$ are the best values.

### Example 3

Letting $\alpha =0.1$, $p = q =2$, to calculate formula (3), we find $\eta ( 0.1 ) = \frac{20 \sqrt[10]{2} \varGamma ( \frac{\pi}{10} )}{\pi \operatorname{csc} ( \frac{\pi}{10} )}=2.253161500^{+}$. Suppose that $f,g\ ( >0 ) \in C_{0.1} ( 0, \infty )$, $0 < {}_{0} I_{\infty}^{0.1} ( f^{2} ( x ) )<\infty$, $0 < {}_{0} I_{\infty}^{0.1} ( g^{2} ( y ) ) <\infty$, then we have the following equivalence inequalities:

13 $$\begin{aligned}& {}_{0} I_{\infty}^{0.1} \biggl[ {}_{0} I_{\infty}^{0.1} \frac{f(x)g(y)}{\max \{ x^{0.1}, y^{0.1} \} } \biggr] < \eta ( 0.1 ) \bigl\{ {}_{0} I_{\infty}^{0.1} \bigl(f^{2} ( x ) \bigr) \bigr\} ^{\frac{1}{2}} \bigl\{ {}_{0} I_{\infty}^{0.1} \bigl( g^{2} ( y ) \bigr) \bigr\} ^{\frac{1}{2}}, \end{aligned}$$

14 $$\begin{aligned}& {}_{0} I_{\infty}^{0.1} \biggl[ {}_{0} I_{\infty}^{0.1} \frac{f(x)}{\max \{ x^{0.1}, y^{n} \} } \biggr]^{2} < \eta^{2} ( 0.1 ) {}_{0} I_{\infty}^{0.1} \bigl( f^{2} ( x ) \bigr), \end{aligned}$$

where the constant factors $\eta ( 0.1 )$, $\eta^{2} (0.1 )$ are the best values.

## Conclusions

In the paper, based on the local fractional calculus theory, a Hilbert-type fractional integral inequality and its equivalent form are tentatively researched. The results show that some methods and skills of the Hilbert-type integral inequality can be transplanted to the research of Hilbert-type fractional integral inequality, which provides a new direction and field to research Hardy-Hilbert’s integral inequalities.
